# Reassortant Avian Influenza Virus (H5N1) in Poultry, Nigeria, 2007

**DOI:** 10.3201/eid1404.071178

**Published:** 2008-04

**Authors:** Isabella Monne, Tony M. Joannis, Alice Fusaro, Paola De Benedictis, Lami H. Lombin, Husseini Ularamu, Anthony Egbuji, Poman Solomon, Tim U. Obi, Giovanni Cattoli, Ilaria Capua

**Affiliations:** * Viale dell’Università, Legnaro, Padova, Italy; † National Veterinary Research Institute, Plateau State, Vom, Nigeria; ‡ University of Ibadan, Oya State, Ibadan, Nigeria; 1 These authors contributed equally to this article

**Keywords:** H5N1, avian influenza, Nigeria, reassortant, phylogenetic analysis, dispatch

## Abstract

Genetic characterization of a selection of influenza virus (H5N1) samples, circulating in 8 Nigerian states over a 39-day period in early 2007, indicates that a new reassortant strain is present in 7 of the 8 states. Our study reports an entirely different influenza virus (H5N1) reassortant becoming predominant and widespread in poultry.

## Abstract 2

Reassortant Influenza Virus (H5N1) in Poultry, Nigeria, 2007

Since its emergence in 2006 in Africa, avian influenza viruses of the H5N1 subtype have spread rapidly to poultry farms in several African countries. In February 2006, Kaduna State in Nigeria was the first of 36 states to report infection of poultry with highly pathogenic avian influenza virus (H5N1). Currently, infection has spread to 22 of the 36 Nigerian states and to the Federal Capital Territory. In February 2007, 1 case of avian influenza was reported in a woman from the southern state of Lagos. Thus, the extensive circulation of influenza virus (H5N1) in Nigeria raises concerns about human and animal health issues. A previous study indicated that 2 sublineages (EMA1 and EMA2) were cocirculating in Nigeria in 2006 (*1*); however, 3 sublineages were identified in a more recent study (*2*), namely sublineage A (corresponding to EMA2) and sublineages B and C (corresponding to EMA1). The 2007 study by Salzberg et al. also identified a virus showing a 4:4 reassortment between genes of sublineages EMA1 and EMA2 (*1*). The aim of our study is to provide additional information on the genetic characteristics of isolates that were circulating in Nigeria in early 2007.

## The Study

Twelve representative influenza virus (H5N1) samples from different Nigerian outbreaks were selected (Table 1) by taking into account the geographic origin and the date of isolation. We then characterized these viruses by sequencing the entire genome.

**Table 1:** List of influenza virus (H5N1) samples analyzed in poultry, Nigeria, 2007

Virus	Group	State of isolation	Date of isolation
A/chicken/Nigeria/1071-1/2007	EMA1/EMA2-2:6-R07	Plateau	Jan 2
A/chicken/Nigeria/1071-3/2007	EMA2	Sokoto	Jan 5
A/chicken/Nigeria/1071-4/2007	EMA1/EMA2-2:6-R07	Borno	Jan 5
A/chicken/Nigeria/1071-5/2007	EMA1/EMA2-2:6–R07	Plateau	Jan 6
A/chicken/Nigeria/1071-7/2007	EMA2	Sokoto	Jan 10
A/chicken/Nigeria/1071-9/2007	EMA1/EMA2-2:6-R07	Bauchi	Jan 12
A/chicken/Nigeria/1071-10/2007	EMA1/EMA2-2:6-R07	Anambra	Jan 13
A/chicken/Nigeria/1071-15/2007	EMA1/EMA2-2:6-R07	Kaduna	Jan 23
A/chicken/Nigeria/1071-22/2007	EMA1/EMA2-2:6-R07	Kano	Jan 31
A/duck/Nigeria/1071-23/2007	EMA1/EMA2-2:6-R07	Borno	Feb 1
A/chicken/Nigeria/1071-29/2007	EMA1/EMA2-2:6-R07	Lagos	Feb 9
A/chicken/Nigeria/1071-30/2007	EMA1/EMA2-2:6-R07	Kaduna	Feb 10

Samples were processed for virus isolation, subtyping, and pathotyping (*3**,**4*). The amplification of the 8 viral gene segments was carried out with reverse transcription (RT)–PCR by using gene-specific primers (available upon request). PCR products were sequenced in a 3100 Avant Genetic Analyzer (Applied Biosystems, Foster City, CA, USA). Phylogenetic analysis was performed by using the neighbor-joining method as implemented in the MEGA 3 program (*5*). GenBank accession nos. for the 8-gene segments of the 12 Nigerian strains are EU148356 to EU148451.

As expected, all Nigerian isolates were closely related to the viruses that have been circulating in birds throughout Europe, Russia, Africa, and the Middle East since late 2005. According to the unified nomenclature system for highly pathogenic influenza virus (H5N1), these isolates belong to clade 2.2 (*6*).

**Figure 1:**
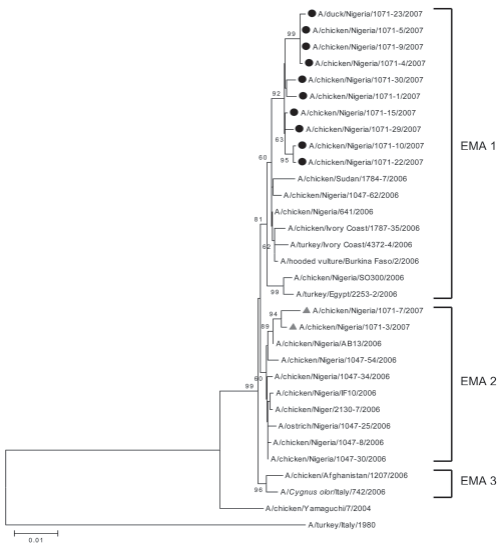
Phylogenetic tree for the hemagglutinin gene of influenza viruses constructed by neighbor-joining method. Sequences obtained in this study were labeled with a circle (EMA1/EMA2-2:6-R07 group) and triangle (EMA2 group). The remaining sequences can be found in GenBank. The numbers at each branch point represent bootstrap values, and they were determined by bootstrap analysis by using 1,000 replications. Scale bar = 0.01 nucleotide substitutions/site.

**Figure 2:**
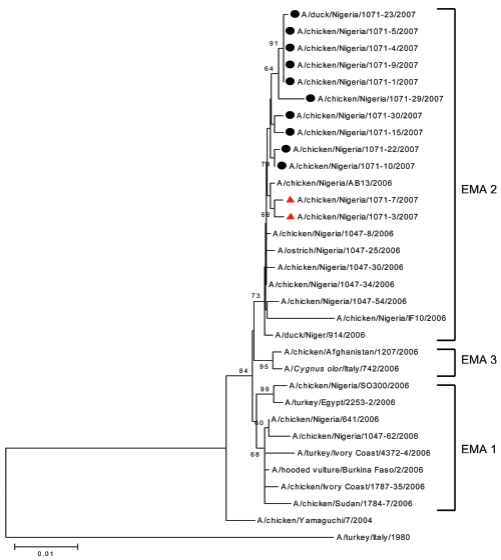
Phylogenetic tree for nucleoprotein gene of influenza viruses constructed by neighbor-joining method. Sequences obtained in this study were labeled with a circle (EMA1/EMA2-2:6-R07 group) and triangle (EMA2 group). The remaining sequences can be found in GenBank. The numbers at each branch point represent bootstrap values, and they were determined by bootstrap analysis by using 1,000 replications. Scale bar = 0.01 nucleotide substitutions/site.

Phylogenetic analysis of all 8 gene segments of the recent Nigerian strains showed that 10 of these strains, labeled EMA1/EMA2-2:6 reassortant 2007 (EMA1/EMA2-2:6-R07) (Table 1), have the same genotype. In particular, the genetic comparison of the hemagglutinin (HA) and nonstructural (NS) genes of EMA1/EMA2-2:6-R07 shows that they are derived from viruses of the EMA1 sublineage (*1*) and have the highest similarity with the first Nigerian strain isolated, A/chicken/Nigeria/641/2006 (homology ranged between 99.3% and 99.7%). We observed different topology for the remaining gene segments (neuraminidase, nucleoprotein [NP], matrix, heterotrimeric polymerase complex [PA, PB1, and PB2]). Phylogenetic analysis showed that the nucleotide sequences of these genes fall into EMA2 sublineage; the highest homology was observed with respective gene segments of the Nigerian strains isolated in 2006, which belong to EMA2 (homology ranged between 99.4% and 99.7%). The separation of the gene segments into 2 clusters (Figures 1, 2) is evidence of reassortment (*7*). The genetic pattern of EMA1/EMA2-2:6-R07 virus is distinct from that of A/chicken/Nigeria/1047-62/2006 virus isolated in June, 2006 in Taraba State and recognized previously as an EMA1/EMA2-4:4 reassortant virus (*1*) (Table 2). The remaining 2 viruses were not reassortants. They were detected in Sokoto State and belong to sublineage EMA2.

**Table 2:** Clustering of the gene segments of influenza strains in poultry, Nigeria, 2007*

Virus	HA	NA	NS	MA	PB2	PB1	PA	NP
A/chicken/Nigeria/641/2006†	EMA1	EMA1	EMA1	EMA1	EMA1	EMA1	EMA1	EMA1
A/chicken/Nigeria/1047-62/2006†	EMA1	EMA2	EMA1	EMA2	EMA2	EMA1	EMA2	EMA1
A/chicken/1071-1/Nigeria/2007 A/chicken/1071-4/Nigeria/2007 A/chicken/1071-5/Nigeria/2007 A/chicken/1071-9/Nigeria/2007 A/chicken/1071-10/Nigeria/2007 A/chicken/1071-15/Nigeria/2007 A/chicken/1071-22/Nigeria/2007 A/duck/1071-23/Nigeria/2007 A/chicken/1071-29/Nigeria/2007 A/chicken/1071-30/Nigeria/2007	EMA1	EMA2	EMA1	EMA2	EMA2	EMA2	EMA2	EMA2
A/chicken/1071-3/Nigeria/2007 A/chicken/1071-7/Nigeria/2007	EMA2	EMA2	EMA2	EMA2	EMA2	EMA2	EMA2	EMA2

*HA, hemagglutinin; NA, neuraminidase; NS, nonstructural; MA; matrix; PB2; polymerase B2; NP, nucleoprotein. †These strains were analyzed in a previous study (*1*).

Sequence analysis showed that the receptor binding site of all of the Nigerian viruses under study retains amino acid residues (Gln 222 and Gly 224). These residues preferentially recognize receptors with saccharides terminating in sialic acid-*α*2-3-galactose (SAα2-3Gal), specific for avian species (*8*). Mutations that are related to neuraminidase inhibitor and adamantanes resistance were not detected in any of the 12 isolates (*9*). All 12 Nigerian viruses possess the PB2 627K mutation associated with increased virulence of influenza virus (H5N1) in mice (*10*). Two other host specific mutations were observed for NP and PA genes. In particular, the NP gene contains a human specific amino acid signature at position 33 (V33I) (*11*) in all selected isolates. A human specific amino acid signature was also detected at position 100 (V100A) of the PA gene of A/chicken/Nigeria/1071-3/2007 and A/chicken/Nigeria/1071-7/2007 strains (*12*).

## Conclusions

The results of our study show that 10 of 12 strains obtained over a 39-day period were EMA1/EMA2-2:6-R07 reassortant viruses, and that these were circulating in at least 7 Nigerian states. This appears to be the only report of a reassortant virus generated by H5N1 viruses belonging to the 2.2 clade spreading extensively in poultry. Thus, the viruses circulating in 2007 in Nigeria differ from the original sublineage prototypes introduced during 2006. Our findings also suggest that an influenza virus (H5N1) with new genetic characteristics has emerged in <7 months and is widespread in Nigeria.

The emergence of at least 2 reassortant viruses in Nigeria shows that co-infection with viruses of different sublineages has occurred presumably in poultry. This evidence is most likely a result of poor biosecurity measures implemented by the poultry industry, particularly the live-bird market system, which is known to facilitate mingling of infected birds. In nonindustrialized countries, live bird market systems sometimes allow birds of different species and of unknown health status to share limited space, often the same cage. Birds in the incubation stage or breeds that show a reduced clinical susceptibility may not appear overtly ill and therefore, may be traded in live bird market systems. The movement of infected birds across neighboring regions could explain the genetic relatedness found between influenza virus (H5N1) isolates obtained from 7 Nigerian states. The predominance of a reassortant virus in Nigeria mimicks the previously reported predominance of the Z genotype virus in Asia, although this genotype is believed to contain internal genes originating from non-H5N1 influenza viruses (*13*). The introduction of influenza virus (H5N1) of different clusters in 2005 in Vietnam, also resulted in the emergence of a reassortant strain. Unlike the Nigerian situation described here, the Vietnamese reassortant influenza virus (H5N1) did not become predominant in Vietnam (*1*).

Currently, the genetic characteristics of the human Nigerian isolate are unavailable. Given that human infection occurs after direct contact with infected poultry, studies need to be performed to establish whether the predominant avian influenza virus (EMA1/EMA2-2:6-R07) has also been responsible for human infection. Amino acid mutations towards increased binding to human receptors, namely N182K, Q192R, Q226L, and G228S substitutions in the HA gene (*14**,**15*), do not seem to have occurred in Nigerian influenza (H5N1) strains to date. However, 2 molecular changes, the 33 NP valine to isoleucine substitution, which is typical of human influenza viruses (*11*), and the 627 PB2 glutamic acid to lysine mutation, which increases virulence in the mammalian host, have been detected in all analyzed strains (*10*).

The results of our study suggest that depopulation and biosecurity measures adopted in Nigeria are not sufficient to prevent the spread of the virus and should be improved. Poultry farmers and smallholder poultry producers (those producing a limited number of poultry) must be educated on appropriate control measures for avian influenza. In addition, isolation and genetic characterization of African influenza virus (H5N1) isolates in a transparent environment, should be promoted and supported so that more information can be gathered on the evolution of this virus in Africa.
