# Erratum: Vol. 14, No. 4

**DOI:** 10.3201/eid1405.074697

**Published:** 2008-05

In the article “Reassortant Avian Influenza Virus (H5N1) in Poultry, Nigeria, 2007” by I. Monne et al., the author affiliations contained errors. **Isabella Monne, Tony M. Joannis, Alice Fusaro, Paola De Benedictis, Giovanni Cattoli, and Ilaria Capua are affiliated with** Istituto Zooprofilattico Sperimentale delle Venezie, Legnaro, Padova, Italy.

We regret any confusion this error may have caused.

